# Molecular tools to capture active neural circuits

**DOI:** 10.3389/fncir.2024.1449459

**Published:** 2024-07-19

**Authors:** Taichi Onishi, Kenzo Hirose, Takeshi Sakaba

**Affiliations:** ^1^Department of Pharmacology, Graduate School of Medicine, The University of Tokyo, Bunkyo City, Bunkyo, Japan; ^2^Graduate School of Brain Science, Doshisha University, Kyotanabe, Kyoto, Japan

**Keywords:** neuronal activity, immediately early gene, activity-dependent tools, molecular tools, neural circuits

## Abstract

To understand how neurons and neural circuits function during behaviors, it is essential to record neuronal activity in the brain *in vivo*. Among the various technologies developed for recording neuronal activity, molecular tools that induce gene expression in an activity-dependent manner have attracted particular attention for their ability to clarify the causal relationships between neuronal activity and behavior. In this review, we summarize recently developed activity-dependent gene expression tools and their potential contributions to the study of neural circuits.

## 1 Introduction

Uncovering the relationship between neural circuit activity and behavior is one of the most critical questions in neuroscience. Although only a small fraction of neurons are activated during a specific behavior or event, identifying and manipulating these active neurons provide valuable insights into how the neural circuits process information and execute actions.

One of the most powerful and prevalent technologies for investigating the relationship between neuronal activity and behavior is real-time imaging of cellular activity using genetically-encoded calcium indicators (GECIs) or genetically encoded voltage indicators (GEVIs) (Grewe and Helmchen, 2009; Grienberger et al., 2022). While this approach has significantly clarified the correlation between the two, its limitations include low time resolution, restricted fields of view, and the inability to conclusively prove causality between neuronal activity and behavior (Lecoq et al., 2019; Grienberger et al., 2022).

To overcome these limitations, tools have been developed to express exogenous genes in activated neurons. These tools enable the detection of individual neurons’ activity across the whole brain with single-cell resolution. Moreover, by manipulating neurons with opsins or Designer Receptors Exclusively Activated by Designer Drugs (DREADD) receptors expressed via these tools, it becomes possible to demonstrate the causal relationships between neural circuit activity and behavior through neuronal manipulation (Mayford, 2014; Josselyn and Tonegawa, 2020).

Several tools have been reported for the activity-dependent expression of exogenous molecules to label and manipulate activated neurons. These tools can mainly be classified into two types: those that utilize transcription systems associated with immediate early genes (IEGs), and those that convert the increase in calcium concentration, which accompanies neuronal activity, into reporter gene expression. This review describes recent techniques for labeling and manipulating activated neurons, primarily focusing on their application in the mammalian brain.

## 2 Advances in activity-dependent gene expressing systems

### 2.1 IEG-based systems

IEGs are a group of genes, including *Arc* and *c-Fos*, which undergo rapid transcription when neurons are activated. IEGs have been widely used as markers of activated neurons, visualized through techniques such as immunostaining. However, visualizing neurons with increased activity in association with a specific behavior for an extended period using IEG expression alone is challenging, due to the transient expression and the short lifetime of IEGs. To overcome this limitation of IEG staining, several methods have been developed to express inducible gene switches, including tetracycline transactivator (tTA) and Cre, under IEG promoters.

#### 2.1.1 TetTag

**Figure 1:**
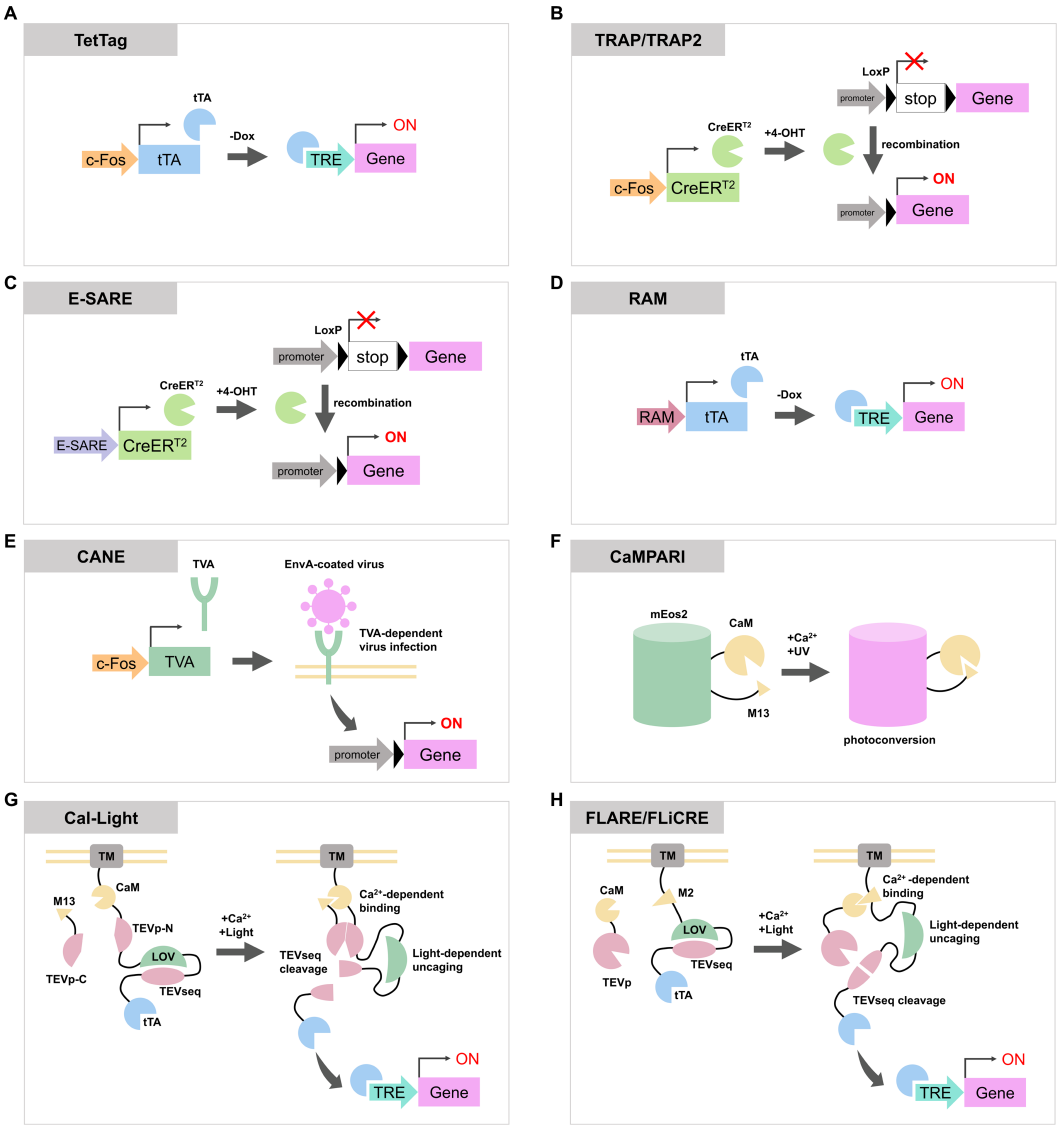
Schematic diagrams of activity-dependent labeling or gene expression tools reviewed in this article. **(A)** In TetTag system, *c-Fos* promoter expresses tTA, and tTA induces a transgene expression when the concentration of Dox is low. **(B)** TRAP and TRAP2 express CreER^T2^ via *c-Fos* promoter. CreER^T2^ recombine LoxP to enable the expression of a reporter gene. **(C,D)** Both E-SARE and RAM are artificial activity-dependent promoters. They can be combined with Tet-OFF system or CreER^T2^ to induce reporter expression. **(E)** In CANE system, activated neurons express destabilized TVA through *c-Fos* promoter. TVA is located on the plasma membrane and allows the entry of EnvA-coated virus containing the reporter gene. This process enables the expression of transgenes in activated neurons. **(F)** CaMPARI photoconverts from the green state to the red state in the presence of UV light and elevated Ca^2+^ concentration. **(G,H)** Cal-Light, FLARE, and FLiCRE are molecular tools with similar designs. When an increase in calcium ion concentration and exposure to blue light occur simultaneously, they cause cleavage of the TEV sequence to release tTA or Cre, inducing the expression of transgenes. tTA, tetracycline transactivator; Dox, doxycycline; TRE, tetracycline response element; 4-OHT, 4-hydroxytamoxifen; TVA, tumor Virus A receptor; EnvA, envelope protein A; CaM, calmodulin; TEVp, tobacco etch virus protease; TEVseq, TEV protease cleavage sequence; LOV, phototropin 1 light-oxygen-voltage 2 domain; TM, transmembrane domain.

In the TetTag system (Reijmers et al., 2007) (Figure 1A), tTA is expressed under the control of an IEG promoter. Upon neuronal activation, the IEG promoter drives the expression of tTA which then binds to the tetracycline response element (TRE) to induce transgene expression. As the presence of doxycycline (Dox) inhibits the binding of tTA to TRE, the concentration of Dox determines the time window for TetTag labeling. Reijmers et al. (2007) used *c-Fos* TetTag mice to label neurons in the basolateral amygdala which were activated during auditory fear conditioning. They demonstrated a strong correlation between the level of fear response during the second exposure to the context or the sound, and the number of IEG expressing neurons during the second exposure which were labeled with TetTag during the first conditioning. TetTag mice have also been used in combination with optogenetic or chemogenetic approaches. Liu et al. (2012) used TetTag mice to express channelrhodopsin-2 only in activated neurons in the dentate gyrus during contextual fear conditioning. By artificially activating these neurons with the light stimulation, mice exhibited freezing behavior, indicating the re-activation of a specific population of dentate gyrus neurons is sufficient for memory retrieval. In addition to these reports, TetTag mice have been widely used for the activity-labeling of neurons in the hippocampus (Ramirez et al., 2013; Tayler et al., 2013; Tanaka et al., 2014; Ohkawa et al., 2015; Okuyama et al., 2016; Pettit et al., 2022), cortex (Stegemann et al., 2023) and spinal cord (Groves et al., 2018).

#### 2.1.2 TRAP and TRAP2

Targeted Recombination in Active Populations (TRAP) expresses tamoxifen-dependent Cre recombinase (CreER^T2^) under the *c-Fos* or *Arc* promoter (Guenthner et al., 2013; DeNardo et al., 2019) (Figure 1B). In this system, when a neuron is activated and tamoxifen is present, CreER^T2^ enter the nucleus and cause recombination of transgenes to enable permanent expression of reporter proteins. The tagging time frame of TRAP is determined by the lifetime of tamoxifen which is approximately one day. Its derivative, 4-hydroxytamoxifen (4-OHT), has a shorter lifetime and TRAP time window of 12 h but maintains the same efficiency as tamoxifen (Guenthner et al., 2013). The first generated TRAP mouse line, also known as TRAP1 or FosTRAP, had CreER^T2^ knocked into the endogenous *c-Fos* loci (Guenthner et al., 2013). Consequently, it could potentially impair the physiological function of Fos. In contrast, TRAP2 has CreER^T2^ fused to the C-terminus of endogenous c-Fos via a 2A peptide. This configuration enables the expression of CreER^T2^ under the regulation of the *c-Fos* promoter while maintaining the expression of c-Fos protein. Additionally, TRAP2 underwent codon optimization for Cre. These upgrades in TRAP2 led to improved labeling efficiency particularly in the striatum, amygdala and hypothalamus (DeNardo et al., 2019). TRAP or TRAP2 mice were also used to label auditory or motor cortex (Tasaka et al., 2018, 2020; Hwang et al., 2022), striatum (Girasole et al., 2018) and hypothalamus (Allen et al., 2017; Ishii et al., 2017). TRAP mice were employed for whole-brain analysis of activated neurons during specific events by crossing them with mice lines that express reporters via Cre recombination (Roy et al., 2022).

#### 2.1.3 E-SARE and RAM

Both enhanced synaptic activity-responsive element (E-SARE) (Figure 1C) and Robust Activity Marking system (RAM) (Figure 1D) are synthetic promoters optimized to enhance efficiency and specificity for activity-dependent labeling. They comprise activity-responsive enhancer modules and minimal promoters of IEGs. E-SARE consists of five tandem repeats of the SARE enhancer region, which recruits activity-regulated transcription factors CREB, MEF2, and SRF (Kawashima et al., 2009), along with the minimal *Arc* promoter. In an assay stimulating cultured neurons, E-SARE has demonstrated higher reporter expression levels and a dynamic range compared to the *c-Fos* promoter (Kawashima et al., 2013). E-SARE was used to label activated neurons in cortex (Kawashima et al., 2013; Cummings et al., 2021) and hippocampus (Attardo et al., 2018).

RAM consists of four tandem repeats of enhancer modules, comprised of a consensus sequence of the Fos/Jun family called AP-1 and a binding motif of the IEG Npas4, and the human *c-Fos* minimal promoter. RAM promoter showed lower basal expression of reporters and a higher dynamic range compared to E-SARE (Sørensen et al., 2016). RAM can be employed in conjunction with the Tet-off expression system to regulate the timing of reporter expression. Sun et al. (2020) replaced enhancer modules of RAM with consensus DNA binding motifs of c-Fos or Npas4 to produce c-Fos-selective RAM (F-RAM) and Npas4-selective RAM (N-RAM). Using N-RAM and F-RAM, they were able to selectively tag ensembles of neurons in which the c-Fos-dependent pathway or Npas4-dependent pathway were enhanced during the contextual fear conditioning. They revealed that synaptic changes received by each ensemble are different, and each ensemble influences opposite roles of memory generalization and discrimination (Sun et al., 2020).

#### 2.1.4 CANE

The capturing activated neuron ensembles (CANE) (Figure 1E) is used by a combination of a knockin mouse line, which contains 2A peptide-dsTVA (destabilized form of avian specific tumor virus A receptor) under the endogenous *c-Fos* loci, and envelope protein A (EnvA)-coated rabies or lentivirus (Sakurai et al., 2016). Activated c-Fos-positive neurons in the knockin mice express dsTVA. dsTVA allows the intracellular entry of EnvA-coated viruses, enabling the introduction of exogenous reporter genes exclusively into activated cells. The short half-life of dsTVA permits control over the timing for reporter expression by adjusting the interval between neuronal activation and the injection of EnvA-coated virus. CANE was used to label c-Fos positive neurons in hypothalamus (Sakurai et al., 2016; Jiang-Xie et al., 2019), periaqueductal grey (Tschida et al., 2019) and pons (Rodriguez et al., 2017). Since CANE can be combined with EnvA-coated rabies virus, it is possible to label presynaptic neurons of the activated neurons through trans-synaptic retrograde infection of the rabies virus (Michael et al., 2020).

### 2.2 Calcium and light dependent systems

IEG promoters and activity-dependent artificial promoters are used for labeling activated neurons, but they suffer from poor temporal resolutions, spanning several hours, and the fact that IEG expression does not always perfectly match cell firing (Fields et al., 1997). To overcome these limitations, systems have been developed in which cell labeling begins only when both calcium elevation and light exposure occur simultaneously. In these systems, experimenters can control the duration of labeling as desired.

#### 2.2.1 CaMPARI

Calcium-modulated photoactivatable ratiometric integrator (CaMPARI) (Figure 1F) is a protein that undergoes photoconversion from the green state to the red state upon simultaneous occurrence of calcium ion concentration elevation and UV irradiation (Fosque et al., 2015). CaMPARI is derived from circularly permuted mEos2 (McKinney et al., 2009), which undergoes irreversible photoconversion upon violet light illumination. To confer calcium responsiveness to the color change, the N- and C-termini of mEos2 were, respectively, fused with the calcium-binding protein calmodulin (CaM) and its target peptide M13. Using CaMPARI, researchers have been able to label neurons dependent on both neuronal activity and UV light. CaMPARI has also been successfully employed in the larval zebrafish brain and the antennal lobe of *Drosophila* (Fosque et al., 2015).

It has been indicated that the photoconversion of CaMPARI occurs even in the absence of calcium ion, and that the efficiency of photoconversion is not high under stimulation conditions. Moeyaert et al. (2018) identified a mutant variant of CaMPARI, named CaMPARI2, through saturation mutagenesis at amino acid positions surrounding the fluorescent protein chromophore and protein interfaces between the fluorescent protein and the calcium-sensitive domains of CaMPARI. This new variant exhibited brighter fluorescence and reduced photoconversion under calcium-free conditions compared to CaMPARI. Although the photoconversion rate under high calcium conditions remained comparable to that of CaMPARI, CaMPARI2 showed slower color change in calcium-free conditions, leading to an improved rate contrast of color change (Moeyaert et al., 2018). Sha et al., (2020) also designed reversibly switchable CaMPARI (rsCaMPARI) which can be switched between bright and dark states. rsCaMPARI. rsCaMPARI shifts from a bright state to a dark state upon calcium binding and blue light exposure, and transitions back to the bright state from the dark state by violet light illumination.

CaMPARI labeling has predominantly been utilized for monitoring color changes in the soma of neurons. However, Perez-Alvarez et al. (2020) introduced SynTagMA, a method designed to localize CaMPARI2 specifically to synapses. By fusing CaMPARI2 to synaptophysin or FingR.PSD95, an intrabody against PSD95 (Gross et al., 2013), they successfully targeted CaMPARI2 to presynaptic and postsynaptic sites, respectively. This enabled them to map synaptic activity.

#### 2.2.2 Cal-Light, FLARE, and FLiCRE

CaMPARI only provides “snapshots” of neurons with increased calcium ion concentration during violet light exposure, and it cannot manipulate the populations of neurons with increased activity using optogenetics or chemogenetics methods. Cal-Light (Lee et al., 2017) and Fast Light- and Activity-Regulated Expression (FLARE) (Wang et al., 2017) are both engineered transcription factors that induce the expression of proteins, including channelrhodopsin, in active neuron populations with high temporal precision (Figures 1G,H).

Cal-Light consists of two components. One component includes a transmembrane (TM) domain, CaM, the N-terminal part of tobacco etch virus (TEV) protease (TEVp-N), a TEV protease cleavage sequence (TEVseq) incorporated into an engineered Jα-helix of phototropin 1 light-oxygen-voltage 2 domains from *Avena sativa* (AsLOV2), and tTA. The other component is a fusion protein of M13 and C-terminal part of TEV protease (TEVp-C). When neurons become active, the concentration of calcium ions in the cytoplasm rises and the binding of calcium ions to CaM in the Cal-Light system increases. This calcium binding triggers a conformational change in CaM, enabling it to interact with M13, which brings TEVp-N and TEVp-C close together. When exposed to the blue light, the AsLOV2 domain undergoes a conformational change to uncover the TEVseq. The proximity of TEVp-N and TEVp-C reconstitutes the active TEV protease, which then cleaves the TEVseq within the AsLOV2 domain. This cleavage releases tTA, enabling the expression of a transgene through the Tet-OFF system in the nucleus (Lee et al., 2017). In the original version of Cal-Light, spontaneous gene expression is occasionally observed. This is attributed to the transient calcium influx at synapses and dendrites independent of action potentials, or the release of stored intracellular calcium. To eliminate the influence of these intrinsic, yet weakly related to neuronal activities, on background gene expression, an improved version called soma targeted Cal-Light (ST-Cal-Light) was developed (Hyun et al., 2022). In ST-Cal-Light, modifications were made to restrict the expression of Cal-Light to the soma, with the aim of minimizing the effect of intrinsic elevations in calcium concentration. The N-terminal 150 residues of the kainate receptor subunit 2, which serve as a soma localization sequence (Shemesh et al., 2017), were inserted between the cytosolic side of the TM domain and CaM (Hyun et al., 2022). In ST-Cal-Light, the expression level remains consistent when both light and calcium are present, while background expression of the reporter gene is suppressed. This resulted in a higher signal-to-noise ratio compared to the original version of Cal-Light in hippocampal primary culture neurons and cortical slices. By utilizing ST-Cal-Light, it was possible to express inhibitory opsins in hippocampal neurons activated during the formation of the contextual fear conditioning and to attenuate freezing responses upon re-exposure to the context (Hyun et al., 2022).

FLARE is a similarly functioning molecular tool consisting of two components. The transcription factor component includes the TM domain of Neurexin3β, the soma targeting sequence of Nav1.6, the CaM binding M2 domain derived from MK2, a TEVseq embedded in an engineered AsLOV2 domain called eLOV, and tTA. The protease component consists of CaM and a truncated TEV protease. Similar to Cal-Light, FLARE utilizes calcium and blue light to release tTA through TEVseq cleavage, which results in the expression of a reporter gene (Wang et al., 2017). As the expression efficiency of FLARE depends on the expression level or ratio of two FLARE components, Sanchez et al. (2020) developed single-chain FLARE (scFLARE). In scFLARE, they replaced CaM and the M2 domain in FLARE with their newly developed calcium-responsive TEV protease (CaTEV) to unite the two FLARE components into a single strand. FLARE and scFLARE were used to label activated neurons in the cortex, amygdala and hippocampus (Wang et al., 2017; Jung et al., 2023; Mocle et al., 2024).

Kim et al. (2020) reported Fast Light and Calcium-Regulated Expression (FLiCRE) which enables more efficient labeling compared to FLARE. In FLiCRE, the TEV protease was replaced with the ultra TEV protease called uTEVp (Sanchez and Ting, 2020), which possesses faster turnover of catalytic activity. Additionally, by combining it with the f-hLOV1, an improved version of LOV domain, obtained through rational engineering of eLOV, the reaction speed was increased, and a higher signal-to-noise ratio was achieved (Kim et al., 2020). It is reported that FLiCRE exhibits fewer leak expression under dark conditions and higher temporal resolution in response to light compared to the original version of Cal-Light (Kim et al., 2020).

## 3 Discussion

We have discussed recent technological advances in labeling and manipulating neurons based on their activity. These tools have made a significant contribution to elucidating the neuronal mechanisms of perception or action. However, there are still issues with these tools. First, particularly with IEG promoter-dependent systems, the temporal resolution of several hours is slower compared to the actual perception or behavior, which occurs within seconds to minutes (Wang et al., 2019). Due to this limitation, it is important to note that there is still a possibility that these neurons do not fire synchronously, although it can be considered that neurons labeled with activity-dependent systems are more likely to fire synchronously than randomly selected neurons. Second, these tools are not universally applicable to all brain regions and all cell types. While many studies have reported using these tools in the hippocampus, it has been noted that the labeling sensitivity of pyramidal neurons in the CA1 and CA3 regions is lower than that of the granule cells in the dentate gyrus when using TetTag or TRAP2 mice (Deng et al., 2013; Leake et al., 2021). Additionally, neurons belonging to functionally distinct circuits tend to express different IEGs (Mahringer et al., 2022).

Tools for activity-dependent labeling or manipulation of neurons so far have been mainly used for research focusing on investigating whether specific groups of neurons existing in particular regions are strongly associated with certain behaviors and whether artificial stimulations and inhibitions are necessary or sufficient for specific behaviors. However, research on how groups of activated neurons are interconnected to form circuits, as well as how the activation of circuits spanning multiple brain regions determines behaviors, has not yet been sufficiently conducted. Understanding how the activity of neural circuits influences behavior serves as an intermediary to explain the correlation between neuronal activity and behavior. In the future, combined with new genetic tools and measurement techniques, activity-dependent gene expression systems will clarify the causal relationship between the activity of neural circuits and behavior.

## Author contributions

TO: Conceptualization, Writing – original draft, Writing – review & editing. KH: Writing – review & editing, Funding acquisition. TS: Funding acquisition, Writing – review & editing.

## References

[ref1] Allen W. E. DeNardo L. A. Chen M. Z. Liu C. D. Loh K. M. Fenno L. E.. (2017). Thirst-associated preoptic neurons encode an aversive motivational drive. Science 357, 1149–1155. doi: 10.1126/science.aan6747, PMID: 28912243 PMC5723384

[ref2] Attardo A. Lu J. Kawashima T. Okuno H. Fitzgerald J. E. Bito H.. (2018). Long-term consolidation of ensemble neural plasticity patterns in hippocampal area CA1. Cell Rep. 25, 640–650.e2. doi: 10.1016/j.celrep.2018.09.064 30332644

[ref3] Cummings K. A. Bayshtok S. Kenny P. J. Clem R. L. (2021). Ensemble encoding of conditioned fear by prefrontal somatostatin interneurons 18, 452791. doi: 10.1101/2021.07.18.452791

[ref4] DeNardo L. A. Liu C. D. Allen W. E. Adams E. L. Friedmann D. Fu L.. (2019). Temporal evolution of cortical ensembles promoting remote memory retrieval. Nat. Neurosci. 22, 460–469. doi: 10.1038/s41593-018-0318-7, PMID: 30692687 PMC6387639

[ref5] Deng W. Mayford M. Gage F. H. (2013). Selection of distinct populations of dentate granule cells in response to inputs as a mechanism for pattern separation in mice. eLife 2:e00312. doi: 10.7554/eLife.00312, PMID: 23538967 PMC3602954

[ref6] Fields R. D. Eshete F. Stevens B. Itoh K. (1997). Action potential-dependent regulation of gene expression: temporal specificity in ca2+, cAMP-responsive element binding proteins, and mitogen-activated protein kinase signaling. J. Neurosci. Off. J. Soc. Neurosci. 17, 7252–7266. doi: 10.1523/JNEUROSCI.17-19-07252.1997, PMID: 9295372 PMC6573446

[ref7] Fosque B. F. Sun Y. Dana H. Yang C.-T. Ohyama T. Tadross M. R.. (2015). Neural circuits labeling of active neural circuits in vivo with designed calcium integrators. Science 347, 755–760. doi: 10.1126/science.1260922, PMID: 25678659

[ref8] Girasole A. E. Lum M. Y. Nathaniel D. Bair-Marshall C. J. Guenthner C. J. Luo L.. (2018). A subpopulation of striatal neurons mediates levodopa-induced dyskinesia. Neuron 97, 787–795.e6. doi: 10.1016/j.neuron.2018.01.017 29398356 PMC6233726

[ref9] Grewe B. F. Helmchen F. (2009). Optical probing of neuronal ensemble activity. Curr. Opin. Neurobiol. 19, 520–529. doi: 10.1016/j.conb.2009.09.003 19854041

[ref10] Grienberger C. Giovannucci A. Zeiger W. Portera-Cailliau C. (2022). Two-photon calcium imaging of neuronal activity. Nat. Rev. Methods Primers. 2:67. doi: 10.1038/s43586-022-00147-1, PMID: 38124998 PMC10732251

[ref11] Gross G. G. Junge J. A. Mora R. J. Kwon H.-B. Olson C. A. Takahashi T. T.. (2013). Recombinant probes for visualizing endogenous synaptic proteins in living neurons. Neuron 78, 971–985. doi: 10.1016/j.neuron.2013.04.017, PMID: 23791193 PMC3779638

[ref12] Groves A. Kihara Y. Jonnalagadda D. Rivera R. Kennedy G. Mayford M.. (2018). A functionally defined in vivo astrocyte population identified by c-Fos activation in a mouse model of multiple sclerosis modulated by S1P dignaling: immediate-early astrocytes (ieAstrocytes). eNeuro 5:182018. doi: 10.1523/ENEURO.0239-18.2018, PMID: 30255127 PMC6153337

[ref13] Guenthner C. J. Miyamichi K. Yang H. H. Heller H. C. Luo L. (2013). Permanent genetic access to transiently active neurons via TRAP: targeted recombination in active populations. Neuron 78, 773–784. doi: 10.1016/j.neuron.2013.03.025, PMID: 23764283 PMC3782391

[ref14] Hwang F.-J. Roth R. H. Wu Y.-W. Sun Y. Kwon D. K. Liu Y.. (2022). Motor learning selectively strengthens cortical and striatal synapses of motor engram neurons. Neuron 110, 2790–2801.e5. doi: 10.1016/j.neuron.2022.06.006, PMID: 35809573 PMC9464700

[ref15] Hyun J. H. Nagahama K. Namkung H. Mignocchi N. Roh S.-E. Hannan P.. (2022). Tagging active neurons by soma-targeted Cal-light. Nat. Commun. 13:7692. doi: 10.1038/s41467-022-35406-y, PMID: 36509775 PMC9744738

[ref16] Ishii K. K. Osakada T. Mori H. Miyasaka N. Yoshihara Y. Miyamichi K.. (2017). A labeled-line neural circuit for pheromone-mediated sexual behaviors in mice. Neuron 95, 123–137.e8. doi: 10.1016/j.neuron.2017.05.038, PMID: 28648498

[ref17] Jiang-Xie L.-F. Yin L. Zhao S. Prevosto V. Han B.-X. Dzirasa K.. (2019). A common neuroendocrine substrate for diverse general anesthetics and sleep. Neuron 102, 1053–1065.e4. doi: 10.1016/j.neuron.2019.03.033, PMID: 31006556 PMC6554048

[ref18] Josselyn S. A. Tonegawa S. (2020). Memory engrams: recalling the past and imagining the future. Science 367:eaaw4325. doi: 10.1126/science.aaw4325, PMID: 31896692 PMC7577560

[ref19] Jung J. H. Wang Y. Rashid A. J. Zhang T. Frankland P. W. Josselyn S. A. (2023). Examining memory linking and generalization using scFLARE2, a temporally precise neuronal activity tagging system. Cell Rep. 42:113592. doi: 10.1016/j.celrep.2023.113592, PMID: 38103203 PMC10842737

[ref20] Kawashima T. Kitamura K. Suzuki K. Nonaka M. Kamijo S. Takemoto-Kimura. (2013). Functional labeling of neurons and their projections using the synthetic activity-dependent promoter E-SARE. Nat. Methods 10, 889–895. doi: 10.1038/nmeth.2559, PMID: 23852453

[ref21] Kawashima T. Okuno H. Nonaka M. Adachi-Morishima A. Kyo N. Okamura M.. (2009). Synaptic activity-responsive element in the arc/Arg3.1 promoter essential for synapse-to-nucleus signaling in activated neurons. Proc. Natl. Acad. Sci. USA 106, 316–321. doi: 10.1073/pnas.0806518106, PMID: 19116276 PMC2629236

[ref22] Kim C. K. Sanchez M. I. Hoerbelt P. Fenno L. E. Malenka R. C. Deisseroth K.. (2020). A molecular calcium integrator reveals a striatal cell type driving aversion. Cell 183, 2003–2019.e16. doi: 10.1016/j.cell.2020.11.015, PMID: 33308478 PMC9839359

[ref23] Leake J. Zinn R. Corbit L. H. Fanselow M. S. Vissel B. (2021). Engram size varies with learning and reflects memory content and precision. J. Neurosci. Off. J. Soc. Neurosci. 41, 4120–4130. doi: 10.1523/JNEUROSCI.2786-20.2021, PMID: 33888604 PMC8176757

[ref24] Lecoq J. Orlova N. Grewe B. F. (2019). Wide. Fast. Deep: recent advances in multiphoton microscopy of in vivo neuronal activity. J. Neurosci. 39, 9042–9052. doi: 10.1523/JNEUROSCI.1527-18.2019, PMID: 31578235 PMC6855689

[ref25] Lee D. Hyun J. H. Jung K. Hannan P. Kwon H.-B. (2017). A calcium- and light-gated switch to induce gene expression in activated neurons. Nat. Biotechnol. 35, 858–863. doi: 10.1038/nbt.3902, PMID: 28650460

[ref26] Liu X. Ramirez S. Pang P. T. Puryear C. B. Govindarajan A. Deisseroth K.. (2012). Optogenetic stimulation of a hippocampal engram activates fear memory recall. Nature 484, 381–385. doi: 10.1038/nature11028, PMID: 22441246 PMC3331914

[ref27] Mahringer D. Zmarz P. Okuno H. Bito H. Keller G. B. (2022). Functional correlates of immediate early gene expression in mouse visual cortex. Peer Community J. 2:e45. doi: 10.24072/pcjournal.156, PMID: 37091727 PMC7614465

[ref28] Mayford M. (2014). The search for a hippocampal engram. Phil. Trans. R. Soc. B 369:20130161. doi: 10.1098/rstb.2013.0161, PMID: 24298162 PMC3843892

[ref29] McKinney S. A. Murphy C. S. Hazelwood K. L. Davidson M. W. Looger L. L. (2009). A bright and photostable photoconvertible fluorescent protein. Nat. Methods 6, 131–133. doi: 10.1038/nmeth.1296, PMID: 19169260 PMC2745648

[ref30] Michael V. Goffinet J. Pearson J. Wang F. Tschida K. Mooney R. (2020). Circuit and synaptic organization of forebrain-to-midbrain pathways that promote and suppress vocalization. eLife 9:e63493. doi: 10.7554/eLife.63493, PMID: 33372655 PMC7793624

[ref31] Mocle A. J. Ramsaran A. I. Jacob A. D. Rashid A. J. Luchetti A. Tran L. M.. (2024). Excitability mediates allocation of pre-configured ensembles to a hippocampal engram supporting contextual conditioned threat in mice. Neuron 112, 1487–1497.e6. doi: 10.1016/j.neuron.2024.02.007, PMID: 38447576 PMC11065628

[ref32] Moeyaert B. Holt G. Madangopal R. Perez-Alvarez A. Fearey B. C. Trojanowski N. F.. (2018). Improved methods for marking active neuron populations. Nat. Commun. 9:4440. doi: 10.1038/s41467-018-06935-2, PMID: 30361563 PMC6202339

[ref33] Ohkawa N. Saitoh Y. Suzuki A. Tsujimura S. Murayama E. Kosugi S.. (2015). Artificial association of pre-stored information to generate a qualitatively new memory. Cell Rep. 11, 261–269. doi: 10.1016/j.celrep.2015.03.017, PMID: 25843716

[ref34] Okuyama T. Kitamura T. Roy D. S. Itohara S. Tonegawa S. (2016). Ventral CA1 neurons store social memory. Science 353, 1536–1541. doi: 10.1126/science.aaf7003, PMID: 27708103 PMC5493325

[ref35] Perez-Alvarez A. Fearey B. C. O’Toole R. J. Yang W. Arganda-Carreras I. Lamothe-Molina P. J.. (2020). Freeze-frame imaging of synaptic activity using SynTagMA. Nat. Commun. 11:2464. doi: 10.1038/s41467-020-16315-4 32424147 PMC7235013

[ref36] Pettit N. L. Yap E.-L. Greenberg M. E. Harvey C. D. (2022). Fos ensembles encode and shape stable spatial maps in the hippocampus. Nature 609, 327–334. doi: 10.1038/s41586-022-05113-1, PMID: 36002569 PMC9452297

[ref37] Ramirez S. Liu X. Lin P.-A. Suh J. Pignatelli M. Redondo R. L.. (2013). Creating a false memory in the hippocampus. Science 341, 387–391. doi: 10.1126/science.1239073, PMID: 23888038

[ref38] Reijmers L. G. Perkins B. L. Matsuo N. Mayford M. (2007). Localization of a stable neural correlate of associative memory. Science 317, 1230–1233. doi: 10.1126/science.1143839, PMID: 17761885

[ref39] Rodriguez E. Sakurai K. Xu J. Chen Y. Toda K. Zhao S.. (2017). A craniofacial-specific monosynaptic circuit enables heightened affective pain. Nat. Neurosci. 20, 1734–1743. doi: 10.1038/s41593-017-0012-1, PMID: 29184209 PMC5819335

[ref40] Roy D. S. Park Y.-G. Kim M. E. Zhang Y. Ogawa S. K. DiNapoli N.. (2022). Brain-wide mapping reveals that engrams for a single memory are distributed across multiple brain regions. Nat. Commun. 13:1799. doi: 10.1038/s41467-022-29384-4, PMID: 35379803 PMC8980018

[ref41] Sakurai K. Zhao S. Takatoh J. Rodriguez E. Lu J. Leavitt A. D.. (2016). Capturing and manipulating activated neuronal ensembles with CANE delineates a hypothalamic social-fear circuit. Neuron 92, 739–753. doi: 10.1016/j.neuron.2016.10.015, PMID: 27974160 PMC5172402

[ref42] Sanchez M. I. Nguyen Q.-A. Wang W. Soltesz I. Ting A. Y. (2020). Transcriptional readout of neuronal activity via an engineered Ca2+—activated protease. Proc. Natl. Acad. Sci. USA 117, 33186–33196. doi: 10.1073/pnas.2006521117, PMID: 33323488 PMC7777206

[ref43] Sanchez M. I. Ting A. Y. (2020). Directed evolution improves the catalytic efficiency of TEV protease. Nat. Methods 17, 167–174. doi: 10.1038/s41592-019-0665-7, PMID: 31819267 PMC7004888

[ref44] Sha F. Abdelfattah A. S. Patel R. Schreiter E. R. (2020). Erasable labeling of neuronal activity using a reversible calcium marker. eLife 9:e57249. doi: 10.7554/eLife.57249, PMID: 32931424 PMC7492087

[ref45] Shemesh O. A. Tanese D. Zampini V. Linghu C. Piatkevich K. Ronzitti E.. (2017). Temporally precise single-cell-resolution optogenetics. Nat. Neurosci. 20, 1796–1806. doi: 10.1038/s41593-017-0018-8, PMID: 29184208 PMC5726564

[ref46] Sørensen A. T. Cooper Y. A. Baratta M. V. Weng F.-J. Zhang Y. Ramamoorthi K.. (2016). A robust activity marking system for exploring active neuronal ensembles. eLife 5:e13918. doi: 10.7554/eLife.13918, PMID: 27661450 PMC5035142

[ref47] Stegemann A. Liu S. Retana Romero O. A. Oswald M. J. Han Y. Beretta C. A.. (2023). Prefrontal engrams of long-term fear memory perpetuate pain perception. Nat. Neurosci. 26, 820–829. doi: 10.1038/s41593-023-01291-x 37024573 PMC10166861

[ref48] Sun X. Bernstein M. J. Meng M. Rao S. Sørensen A. T. Yao L.. (2020). Functionally distinct neuronal ensembles within the memory engram. Cell 181, 410–423.e17. doi: 10.1016/j.cell.2020.02.055, PMID: 32187527 PMC7166195

[ref49] Tanaka K. Z. Pevzner A. Hamidi A. B. Nakazawa Y. Graham J. Wiltgen B. J. (2014). Cortical representations are reinstated by the hippocampus during memory retrieval. Neuron 84, 347–354. doi: 10.1016/j.neuron.2014.09.037 25308331

[ref50] Tasaka G.-I. Feigin L. Maor I. Groysman M. DeNardo L. A. Schiavo J. K.. (2020). The temporal association cortex plays a key role in auditory-driven maternal plasticity. Neuron 107, 566–579.e7. doi: 10.1016/j.neuron.2020.05.004, PMID: 32473095 PMC8725613

[ref51] Tasaka G.-I. Guenthner C. J. Shalev A. Gilday O. Luo L. Mizrahi A. (2018). Genetic tagging of active neurons in auditory cortex reveals maternal plasticity of coding ultrasonic vocalizations. Nat. Commun. 9:871. doi: 10.1038/s41467-018-03183-2, PMID: 29491360 PMC5830453

[ref52] Tayler K. K. Tanaka K. Z. Reijmers L. G. Wiltgen B. J. (2013). Reactivation of neural ensembles during the retrieval of recent and remote memory. Curr. Biol. 23, 99–106. doi: 10.1016/j.cub.2012.11.019, PMID: 23246402

[ref53] Tschida K. Michael V. Takatoh J. Han B.-X. Zhao S. Sakurai K.. (2019). A specialized neural circuit gates social vocalizations in the mouse. Neuron 103, 459–472.e4. doi: 10.1016/j.neuron.2019.05.025, PMID: 31204083 PMC6687542

[ref54] Wang W. Kim C. K. Ting A. Y. (2019). Molecular tools for imaging and recording neuronal activity. Nat. Chem. Biol. 15, 101–110. doi: 10.1038/s41589-018-0207-0 30659298

[ref55] Wang W. Wildes C. P. Pattarabanjird T. Sanchez M. I. Glober G. F. Matthews G. A.. (2017). A light- and calcium-gated transcription factor for imaging and manipulating activated neurons. Nat. Biotechnol. 35, 864–871. doi: 10.1038/nbt.3909, PMID: 28650461 PMC5595644

